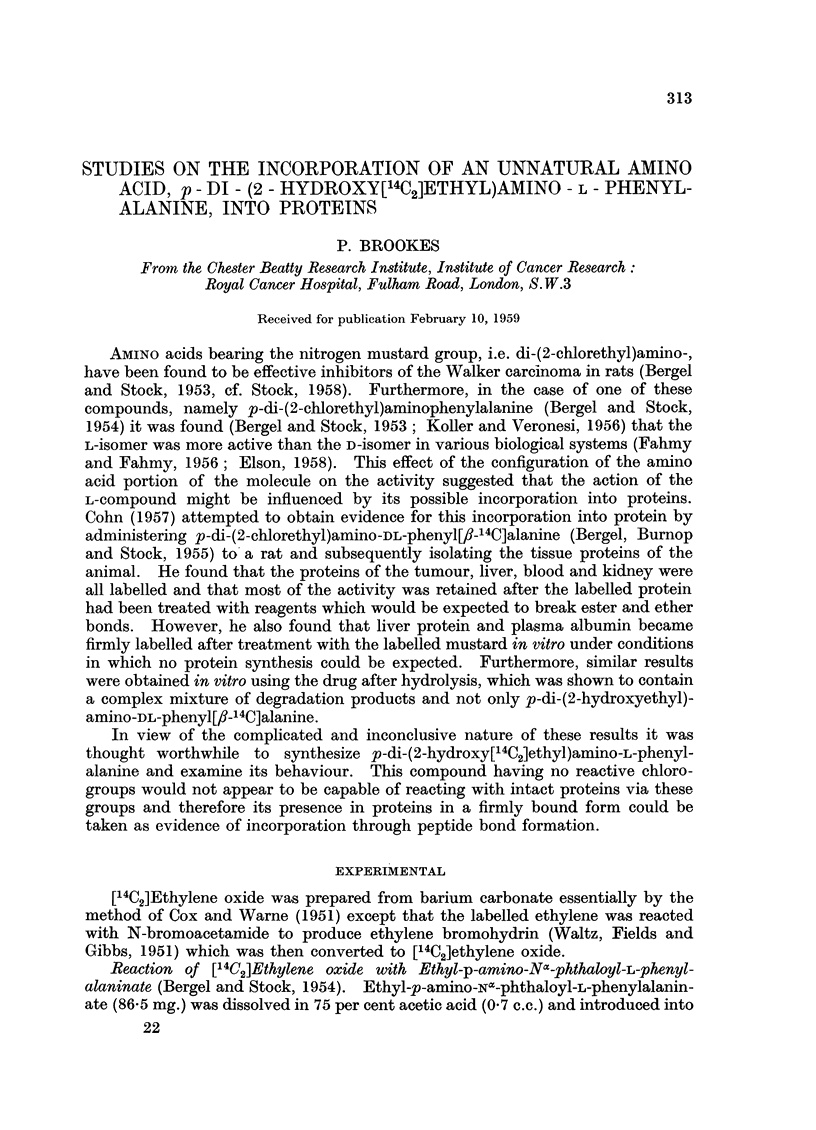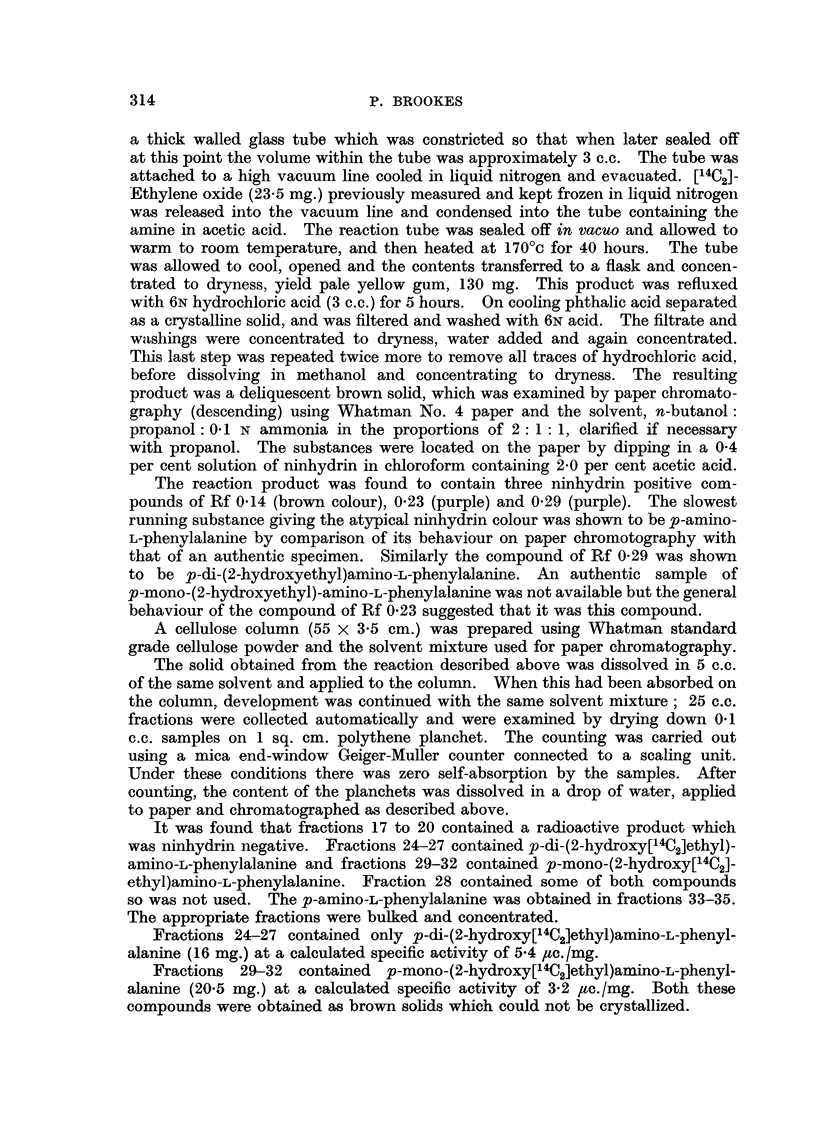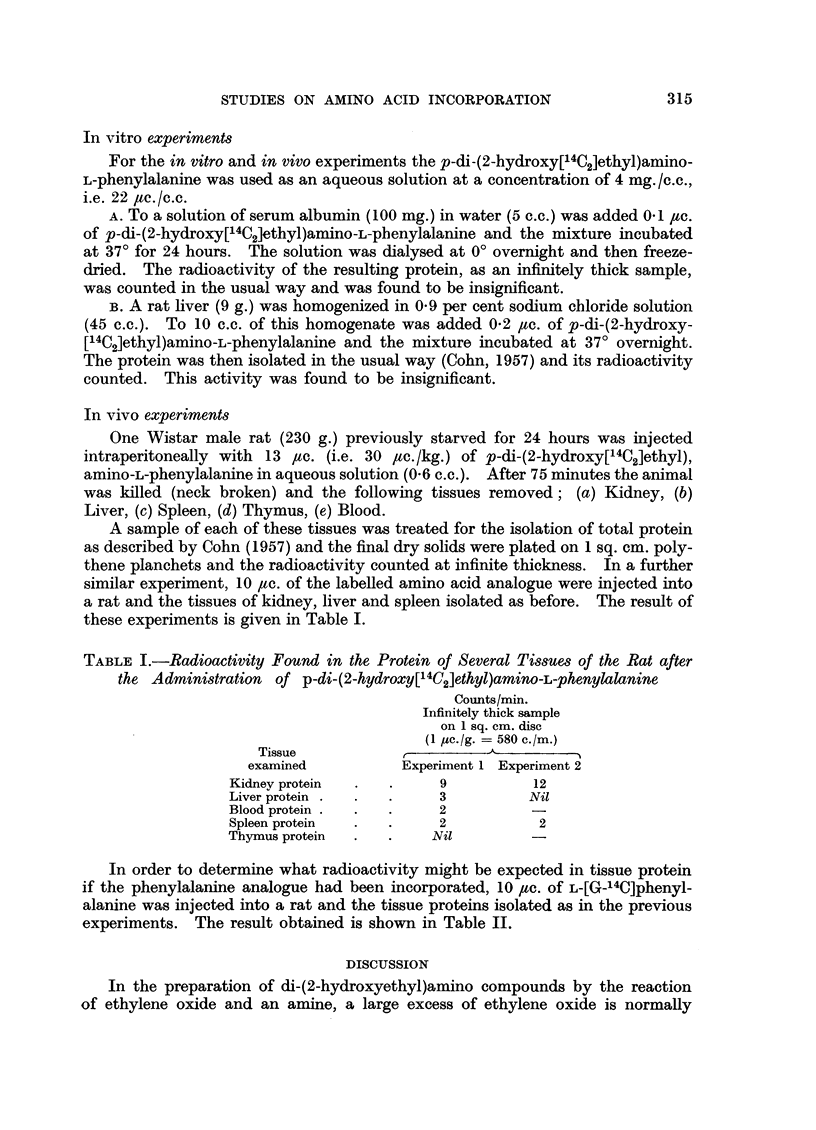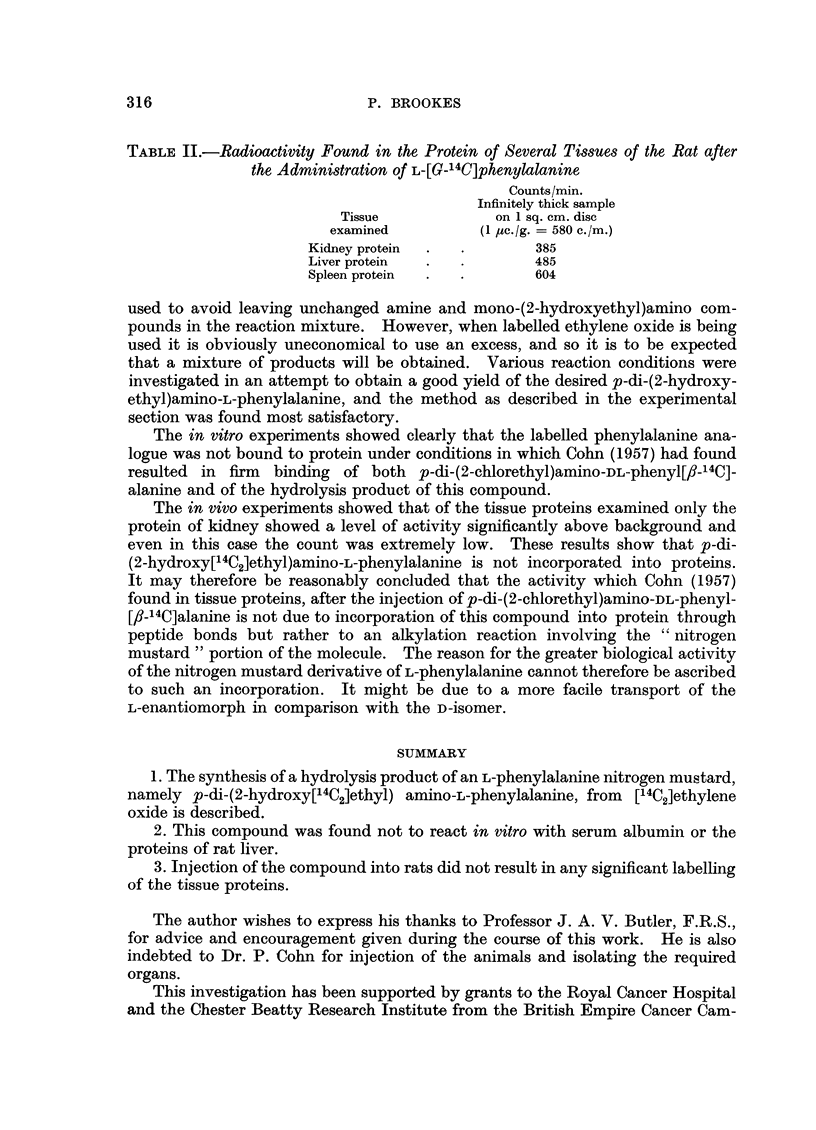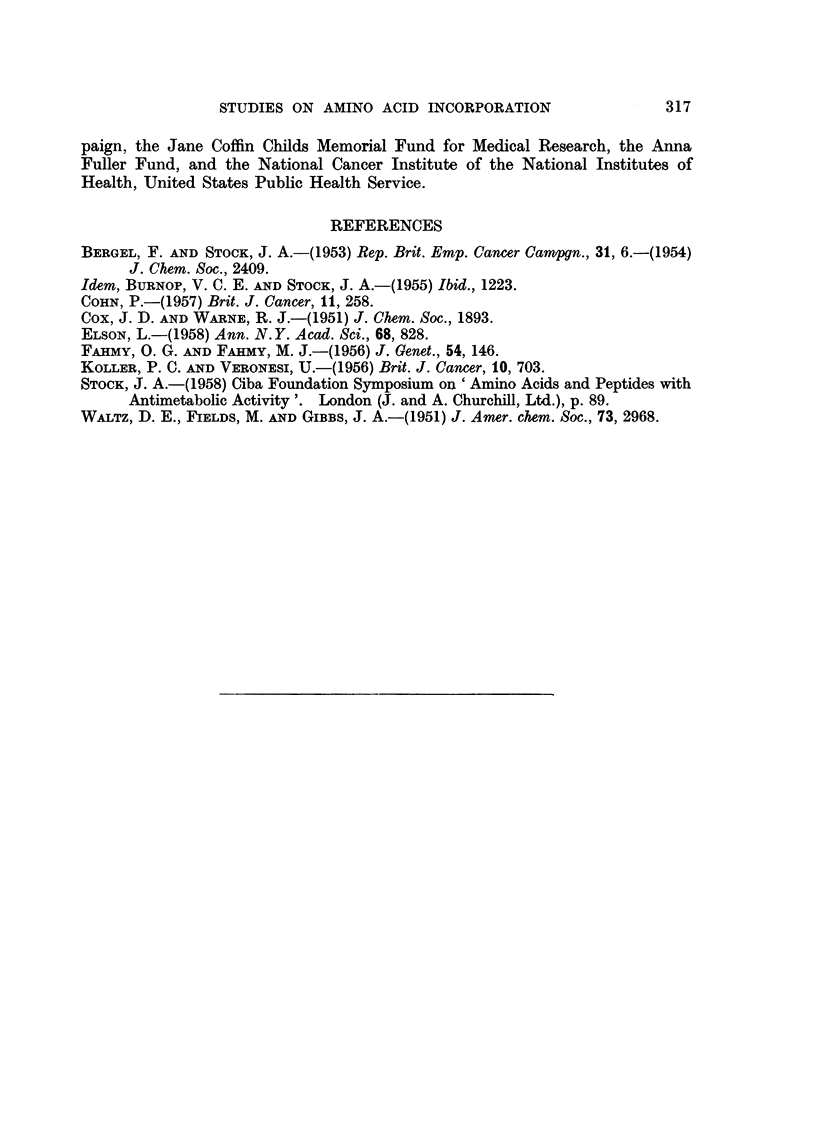# Studies on the Incorporation of an Unnatural Amino Acid, p-DI-(2-Hydroxy[^14^C_2_]Ethyl) Amino-L-Phenylalanine, into Proteins

**DOI:** 10.1038/bjc.1959.38

**Published:** 1959-06

**Authors:** P. Brookes


### STUDIES ON THE INCORPORATION OF AN UNNATURAL AMINO ACID, *p*-DI-(2-HYDROXY[$^{14}C_2$]ETHYL)AMINO-L-PHENYLALANINE, INTO PROTEINS

P. BROOKES

*From the Chester Beatty Research Institute, Institute of Cancer Research: Royal Cancer Hospital, Fulham Road, London, S.W.3*

Received for publication February 10, 1959

AMINO acids bearing the nitrogen mustard group, i.e. di-(2-chlorethyl)amino-, have been found to be effective inhibitors of the Walker carcinoma in rats (Bergel and Stock, 1953, cf. Stock, 1958). Furthermore, in the case of one of these compounds, namely *p*-di-(2-chlorethyl)aminophenylalanine (Bergel and Stock, 1954) it was found (Bergel and Stock, 1953; Koller and Veronesi, 1956) that the L-isomer was more active than the D-isomer in various biological systems (Fahmy and Fahmy, 1956; Elson, 1958). This effect of the configuration of the amino acid portion of the molecule on the activity suggested that the action of the L-compound might be influenced by its possible incorporation into proteins. Cohn (1957) attempted to obtain evidence for this incorporation into protein by administering *p*-di-(2-chlorethyl)amino-DL-phenyl[$\beta$-$^{14}$C]alanine (Bergel, Burnop and Stock, 1955) to a rat and subsequently isolating the tissue proteins of the animal. He found that the proteins of the tumour, liver, blood and kidney were all labelled and that most of the activity was retained after the labelled protein had been treated with reagents which would be expected to break ester and ether bonds. However, he also found that liver protein and plasma albumin became firmly labelled after treatment with the labelled mustard *in vitro* under conditions in which no protein synthesis could be expected. Furthermore, similar results were obtained *in vitro* using the drug after hydrolysis, which was shown to contain a complex mixture of degradation products and not only *p*-di-(2-hydroxyethyl)-amino-DL-phenyl[$\beta$-$^{14}$C]alanine.

In view of the complicated and inconclusive nature of these results it was thought worthwhile to synthesize *p*-di-(2-hydroxy[$^{14}C_2$]ethyl)amino-L-phenylalanine and examine its behaviour. This compound having no reactive chloro-groups would not appear to be capable of reacting with intact proteins via these groups and therefore its presence in proteins in a firmly bound form could be taken as evidence of incorporation through peptide bond formation.

### EXPERIMENTAL

[$^{14}C_2$]Ethylene oxide was prepared from barium carbonate essentially by the method of Cox and Warne (1951) except that the labelled ethylene was reacted with N-bromoacetamide to produce ethylene bromohydrin (Waltz, Fields and Gibbs, 1951) which was then converted to [$^{14}C_2$]ethylene oxide.

*Reaction of [$^{14}C_2$]Ethylene oxide with Ethyl-p-amino-N$^{\alpha}$-phthaloyl-L-phenylalaninate* (Bergel and Stock, 1954). Ethyl-*p*-amino-N$^{\alpha}$-phthaloyl-L-phenylalaninate (86·5 mg.) was dissolved in 75 per cent acetic acid (0·7 c.c.) and introduced into a thick walled glass tube which was constricted so that when later sealed off at this point the volume within the tube was approximately 3 c.c. The tube was attached to a high vacuum line cooled in liquid nitrogen and evacuated. [$^{14}C_2$]-Ethylene oxide (23·5 mg.) previously measured and kept frozen in liquid nitrogen was released into the vacuum line and condensed into the tube containing the amine in acetic acid. The reaction tube was sealed off *in vacuo* and allowed to warm to room temperature, and then heated at 170°C for 40 hours. The tube was allowed to cool, opened and the contents transferred to a flask and concentrated to dryness, yield pale yellow gum, 130 mg. This product was refluxed with 6N hydrochloric acid (3 c.c.) for 5 hours. On cooling phthalic acid separated as a crystalline solid, and was filtered and washed with 6N acid. The filtrate and washings were concentrated to dryness, water added and again concentrated. This last step was repeated twice more to remove all traces of hydrochloric acid, before dissolving in methanol and concentrating to dryness. The resulting product was a deliquescent brown solid, which was examined by paper chromatography (descending) using Whatman No. 4 paper and the solvent, *n*-butanol : propanol : 0·1 N ammonia in the proportions of 2 : 1 : 1, clarified if necessary with propanol. The substances were located on the paper by dipping in a 0·4 per cent solution of ninhydrin in chloroform containing 2·0 per cent acetic acid.

The reaction product was found to contain three ninhydrin positive compounds of Rf 0·14 (brown colour), 0·23 (purple) and 0·29 (purple). The slowest running substance giving the atypical ninhydrin colour was shown to be *p*-amino-L-phenylalanine by comparison of its behaviour on paper chromotography with that of an authentic specimen. Similarly the compound of Rf 0·29 was shown to be *p*-di-(2-hydroxyethyl)amino-L-phenylalanine. An authentic sample of *p*-mono-(2-hydroxyethyl)-amino-L-phenylalanine was not available but the general behaviour of the compound of Rf 0·23 suggested that it was this compound.

A cellulose column (55 × 3·5 cm.) was prepared using Whatman standard grade cellulose powder and the solvent mixture used for paper chromatography.

The solid obtained from the reaction described above was dissolved in 5 c.c. of the same solvent and applied to the column. When this had been absorbed on the column, development was continued with the same solvent mixture ; 25 c.c. fractions were collected automatically and were examined by drying down 0·1 c.c. samples on 1 sq. cm. polythene planchet. The counting was carried out using a mica end-window Geiger-Muller counter connected to a scaling unit. Under these conditions there was zero self-absorption by the samples. After counting, the content of the planchets was dissolved in a drop of water, applied to paper and chromatographed as described above.

It was found that fractions 17 to 20 contained a radioactive product which was ninhydrin negative. Fractions 24–27 contained *p*-di-(2-hydroxy[$^{14}C_2$]ethyl)-amino-L-phenylalanine and fractions 29–32 contained *p*-mono-(2-hydroxy[$^{14}C_2$]-ethyl)amino-L-phenylalanine. Fraction 28 contained some of both compounds so was not used. The *p*-amino-L-phenylalanine was obtained in fractions 33–35. The appropriate fractions were bulked and concentrated.

Fractions 24–27 contained only *p*-di-(2-hydroxy[$^{14}C_2$]ethyl)amino-L-phenylalanine (16 mg.) at a calculated specific activity of 5·4 μc./mg.

Fractions 29–32 contained *p*-mono-(2-hydroxy[$^{14}C_2$]ethyl)amino-L-phenylalanine (20·5 mg.) at a calculated specific activity of 3·2 μc./mg. Both these compounds were obtained as brown solids which could not be crystallized.

In vitro *experiments*

For the *in vitro* and *in vivo* experiments the *p*-di-(2-hydroxy[$^{14}C_2$]ethyl)amino-L-phenylalanine was used as an aqueous solution at a concentration of 4 mg./c.c., i.e. 22 $\mu$c./c.c.

A. To a solution of serum albumin (100 mg.) in water (5 c.c.) was added 0·1 $\mu$c. of *p*-di-(2-hydroxy[$^{14}C_2$]ethyl)amino-L-phenylalanine and the mixture incubated at 37° for 24 hours. The solution was dialysed at 0° overnight and then freeze-dried. The radioactivity of the resulting protein, as an infinitely thick sample, was counted in the usual way and was found to be insignificant.

B. A rat liver (9 g.) was homogenized in 0·9 per cent sodium chloride solution (45 c.c.). To 10 c.c. of this homogenate was added 0·2 $\mu$c. of *p*-di-(2-hydroxy-[$^{14}C_2$]ethyl)amino-L-phenylalanine and the mixture incubated at 37° overnight. The protein was then isolated in the usual way (Cohn, 1957) and its radioactivity counted. This activity was found to be insignificant.

In vivo *experiments*

One Wistar male rat (230 g.) previously starved for 24 hours was injected intraperitoneally with 13 $\mu$c. (i.e. 30 $\mu$c./kg.) of *p*-di-(2-hydroxy[$^{14}C_2$]ethyl), amino-L-phenylalanine in aqueous solution (0·6 c.c.). After 75 minutes the animal was killed (neck broken) and the following tissues removed; (*a*) Kidney, (*b*) Liver, (*c*) Spleen, (*d*) Thymus, (*e*) Blood.

A sample of each of these tissues was treated for the isolation of total protein as described by Cohn (1957) and the final dry solids were plated on 1 sq. cm. polythene planchets and the radioactivity counted at infinite thickness. In a further similar experiment, 10 $\mu$c. of the labelled amino acid analogue were injected into a rat and the tissues of kidney, liver and spleen isolated as before. The result of these experiments is given in Table I.

TABLE I.—*Radioactivity Found in the Protein of Several Tissues of the Rat after the Administration of* p-*di-(2-hydroxy[$^{14}C_2$]ethyl)amino-*L-*phenylalanine*

| Tissue examined | Counts/min. Infinitely thick sample on 1 sq. cm. disc (1 $\mu$c./g. = 580 c./m.) | |
|---|---|---|
| | Experiment 1 | Experiment 2 |
| Kidney protein . . | 9 | 12 |
| Liver protein . . . | 3 | *Nil* |
| Blood protein . . . | 2 | — |
| Spleen protein . . | 2 | 2 |
| Thymus protein . . | *Nil* | — |

In order to determine what radioactivity might be expected in tissue protein if the phenylalanine analogue had been incorporated, 10 $\mu$c. of L-[G-$^{14}$C]phenylalanine was injected into a rat and the tissue proteins isolated as in the previous experiments. The result obtained is shown in Table II.

### DISCUSSION

In the preparation of di-(2-hydroxyethyl)amino compounds by the reaction of ethylene oxide and an amine, a large excess of ethylene oxide is normally

TABLE II.—*Radioactivity Found in the Protein of Several Tissues of the Rat after the Administration of* L-[*G*-$^{14}$*C*]*phenylalanine*

| Tissue examined | Counts/min. Infinitely thick sample on 1 sq. cm. disc (1 $\mu$c./g. = 580 c./m.) |
|---|---|
| Kidney protein . . | 385 |
| Liver protein . . | 485 |
| Spleen protein . . | 604 |

used to avoid leaving unchanged amine and mono-(2-hydroxyethyl)amino compounds in the reaction mixture. However, when labelled ethylene oxide is being used it is obviously uneconomical to use an excess, and so it is to be expected that a mixture of products will be obtained. Various reaction conditions were investigated in an attempt to obtain a good yield of the desired *p*-di-(2-hydroxyethyl)amino-L-phenylalanine, and the method as described in the experimental section was found most satisfactory.

The *in vitro* experiments showed clearly that the labelled phenylalanine analogue was not bound to protein under conditions in which Cohn (1957) had found resulted in firm binding of both *p*-di-(2-chlorethyl)amino-DL-phenyl[$\beta$-$^{14}$C]-alanine and of the hydrolysis product of this compound.

The *in vivo* experiments showed that of the tissue proteins examined only the protein of kidney showed a level of activity significantly above background and even in this case the count was extremely low. These results show that *p*-di-(2-hydroxy[$^{14}C_2$]ethyl)amino-L-phenylalanine is not incorporated into proteins. It may therefore be reasonably concluded that the activity which Cohn (1957) found in tissue proteins, after the injection of *p*-di-(2-chlorethyl)amino-DL-phenyl-[$\beta$-$^{14}$C]alanine is not due to incorporation of this compound into protein through peptide bonds but rather to an alkylation reaction involving the " nitrogen mustard " portion of the molecule. The reason for the greater biological activity of the nitrogen mustard derivative of L-phenylalanine cannot therefore be ascribed to such an incorporation. It might be due to a more facile transport of the L-enantiomorph in comparison with the D-isomer.

### SUMMARY

1. The synthesis of a hydrolysis product of an L-phenylalanine nitrogen mustard, namely *p*-di-(2-hydroxy[$^{14}C_2$]ethyl) amino-L-phenylalanine, from [$^{14}C_2$]ethylene oxide is described.

2. This compound was found not to react *in vitro* with serum albumin or the proteins of rat liver.

3. Injection of the compound into rats did not result in any significant labelling of the tissue proteins.

The author wishes to express his thanks to Professor J. A. V. Butler, F.R.S., for advice and encouragement given during the course of this work. He is also indebted to Dr. P. Cohn for injection of the animals and isolating the required organs.

This investigation has been supported by grants to the Royal Cancer Hospital and the Chester Beatty Research Institute from the British Empire Cancer Campaign, the Jane Coffin Childs Memorial Fund for Medical Research, the Anna Fuller Fund, and the National Cancer Institute of the National Institutes of Health, United States Public Health Service.

### REFERENCES

BERGEL, F. AND STOCK, J. A.—(1953) *Rep. Brit. Emp. Cancer Campgn.*, **31**, 6.—(1954) *J. Chem. Soc.*, 2409.

*Idem*, BURNOP, V. C. E. AND STOCK, J. A.—(1955) *Ibid.*, 1223.

COHN, P.—(1957) *Brit. J. Cancer*, **11**, 258.

COX, J. D. AND WARNE, R. J.—(1951) *J. Chem. Soc.*, 1893.

ELSON, L.—(1958) *Ann. N.Y. Acad. Sci.*, **68**, 828.

FAHMY, O. G. AND FAHMY, M. J.—(1956) *J. Genet.*, **54**, 146.

KOLLER, P. C. AND VERONESI, U.—(1956) *Brit. J. Cancer*, **10**, 703.

STOCK, J. A.—(1958) Ciba Foundation Symposium on ' Amino Acids and Peptides with Antimetabolic Activity '. London (J. and A. Churchill, Ltd.), p. 89.

WALTZ, D. E., FIELDS, M. AND GIBBS, J. A.—(1951) *J. Amer. chem. Soc.*, **73**, 2968.